# Effects of hypoxia on antigen presentation and T cell-based immune recognition of HPV16-transformed cells

**DOI:** 10.3389/fimmu.2022.918528

**Published:** 2022-10-21

**Authors:** Nitya Mohan, Kathrin Wellach, Ceren Özerdem, Nisha Veits, Jonas D. Förster, Sophia Foehr, Maria Bonsack, Angelika B. Riemer

**Affiliations:** ^1^ Immunotherapy and Immunoprevention, German Cancer Research Center (DKFZ), Heidelberg, Germany; ^2^ Molecular Vaccine Design, German Center for Infection Research (DZIF), Partner Site Heidelberg, Heidelberg, Germany; ^3^ Faculty of Biosciences, Heidelberg University, Heidelberg, Germany

**Keywords:** Hypoxia, human papillomavirus (HPV), antigen processing and presentation machinery (APM), cytotoxic T cells, tumor microenvironment (TME)

## Abstract

Attempts to develop a therapeutic vaccine against human papillomavirus (HPV)-induced malignancies have mostly not been clinically successful to date. One reason may be the hypoxic microenvironment present in most tumors, including cervical cancer. Hypoxia dysregulates the levels of human leukocyte antigen (HLA) class I molecules in different tumor entities, impacts the function of cytotoxic T cells, and leads to decreased protein levels of the oncoproteins E6 and E7 in HPV-transformed cells. Therefore, we investigated the effect of hypoxia on the presentation of HPV16 E6- and E7-derived epitopes in cervical cancer cells and its effect on epitope-specific T cell cytotoxicity. Hypoxia induced downregulation of E7 protein levels in all analyzed cell lines, as assessed by Western blotting. However, contrary to previous reports, no perturbation of antigen processing and presentation machinery (APM) components and HLA-A2 surface expression upon hypoxia treatment was detected by mass spectrometry and flow cytometry, respectively. Cytotoxicity assays performed in hypoxic conditions showed differential effects on the specific killing of HPV16-positive cervical cancer cells by epitope-specific CD8^+^ T cell lines in a donor- and peptide-specific manner. Effects of hypoxia on the expression of PD-L1 were ruled out by flow cytometry analysis. Altogether, our results under hypoxia show a decreased expression of E6 and E7, but an intact APM, and epitope- and donor-dependent effects on T cell cytotoxicity towards HPV16-positive target cells. This suggests that successful immunotherapies can be developed for hypoxic HPV-induced cervical cancer, with careful choice of target epitopes, and ideally in combination with hypoxia-alleviating measures.

## Introduction

High-risk human papillomaviruses (HPVs) are responsible for cervical cancers, and a major proportion of other anogenital and oropharyngeal cancers (1). As of 2018, cervical cancer was the fourth most common cancer in women worldwide and caused >300,000 deaths annually (2). HPV16 alone is responsible for about 60% of cervical cancer cases worldwide (3). These cancers are thought to be the perfect ground for the development of targeted immunotherapeutic strategies (4). Being virally induced, they provide a repertoire of immunologically foreign cancer-specific antigens as ideal therapeutic targets. In particular, the viral oncoproteins E6 and E7 are considered to be promising targets as they are essential for malignant transformation, which does not allow tumor escape by antigen loss (5). Moreover, effective immune responses against HPV associated with virus clearance are mounted by most infected individuals. This proves that the immune system is capable of recognizing HPV-derived antigens and inducing immunity (6). For patients having developed a persistent infection or malignancy, therapeutic options involve surgery and/or chemo- and radiotherapy with associated risks and tissue damage. Therefore, a variety of immunotherapy approaches against HPV have been developed and tested with partly promising results in clinical trials (7, 8). However, there are no approved therapeutic HPV vaccines as of yet. This suggests that there is a need to elucidate other aspects of cervical cancer pathophysiology before a successful therapeutic vaccine can be developed. One important feature, which has only recently received attention, is the tumor microenvironment (TME) (9).

The components of the TME comprise the tissue surrounding and interacting with the tumor such as extracellular matrix (ECM), vasculature, stromal cells, and immune cells, as well as the oxygen concentration and pH levels (10). Together, these factors influence cancer progression in several ways (11). A key factor that regulates other TME aspects and affects all the hallmarks of cancer is hypoxia (12). Hypoxia develops as a proliferating tumor outgrows its surrounding vasculature leading to the reduction of oxygen concentration below 2% (13). This induces the transcription factor hypoxia-inducible factor 1 α (HIF1α), which is a key regulator of hypoxia-modified pathways. HIF1α regulates several downstream pathways, including other transcription factors (14). Of significant interest in the context of immunotherapies is the immunosuppressive effect of the hypoxic TME, aiding the tumor’s escape from immune surveillance (15). The effect of hypoxia on different immune cell types has been elaborately investigated (16). In particular, the impact on cytotoxic T cells (CD8^+^ T cells) is of pertinence from the perspective of therapeutic vaccine design. It has been reported that exposure of CD8^+^ tumor-infiltrating lymphocytes (TILs) to hypoxia led to their decreased proliferation. Moreover, these hypoxic TILs secreted the immunosuppressive cytokine IL-10 (17). However, counterintuitively, a recent study has demonstrated that CD8^+^ T cells pre-cultured in hypoxia led to a higher tumor rejection and improved survival in B16-OVA tumor-bearing mice than normoxic CD8^+^ T cells (18). This enhancement of anti-tumor responses by hypoxia warrants further investigation.

To be recognized by CD8^+^ T cells, antigens need to be processed by the antigen processing and presentation machinery (APM) and presented on the cell surface in complex with a major histocompatibility complex class I (MHC I) molecule. This aspect has been reported to be regulated by hypoxia in a cancer type-specific manner. Some studies have shown an upregulation of the human MHC (human leukocyte antigen, HLA) I in melanoma and colorectal cancer cell lines (19, 20). Another study demonstrated downregulation of MHC I and other APM components (TAP1, TAP2, PSMB9, and PSMB8) *in vitro* as well as *in vivo* using 3D culture systems as well as sarcoma and pulmonary tumor mouse models (21). Regulation of immune cell functions and antigen presentation by hypoxic TME have prompted an investigation into its effect on immunotherapies. Vaccination with cell lysates generated under 5% O_2_ was observed to lead to enhanced cytolytic function of CD8^+^ T cells in mouse models of breast cancer and glioma (22). However, the effect of hypoxia on immunotherapies of cervical cancers has not been investigated yet. Research has shown that hypoxia induces a state of dormancy in cervical cancer cells, which was reversible upon reoxygenation. Mechanistically, this was due to a downregulation of E6 and E7 oncoproteins at the transcriptional level, *via* the regulation of the PI3K/mTORC2/Akt axis, selectively in hypoxic HPV-transformed cells (23–25). As E6 and E7 are the targets of most therapeutic HPV vaccination approaches, their reported downregulation under hypoxia at both protein and mRNA levels (23) is a cause of concern. We hypothesized that the TME, particularly hypoxia, is responsible for limiting the efficiency of HPV-targeted immunotherapies. Thus, we investigated the hypoxia-induced effects on E6 and E7 and the APM in a comprehensive panel of HPV16-positive cancer cell lines and on target cell lysis mediated by HPV16-epitope-specific CD8^+^ T cells.

## Materials and methods

### Hypoxic and normoxic cultivation of cervical cancer and control cell lines

HPV16-positive cervical cancer and control cell lines were cultured in their corresponding media (**Supplementary Table 1**). CaSki and C33A were obtained from ATCC (CRL-1550 and HTB-31, respectively). SNU17, SNU703, and SNU1299 were obtained from the Korean Cell Line Bank. 866 was kindly provided by P. L. Stern (University of Manchester, UK), MRI-H-196 by E. Schwarz (German Cancer Research Center, Heidelberg, Germany), Marqu by A. Kaufmann (Charité, Berlin, Germany), and NOK by K. Münger (Tufts University, Boston, MA, USA). Cell lines were regularly authenticated and confirmed to be free of Mycoplasma, SMRV, or interspecies contamination by SNP profiling and multiplex-PCR by Multiplexion GmbH (26). At a confluence of 70%, biological replicates were simultaneously treated with either hypoxia or normoxia. For hypoxia treatment, cells were placed in an InVivo2 workstation (Baker Ruskinn) at 1% O_2_ and 5% CO_2_, for the duration as indicated in respective figures. Hypoxia treatment was performed in low-glucose conditions, by a medium change to the corresponding low-glucose medium 24 h prior to treatment (**Supplementary Table 1**). Harvesting of cells was performed using cell culture grade trypsin (Sigma-Aldrich). Briefly, after washing with 1× PBS, the cells were detached by incubation with trypsin for 5–7 min. Subsequently, FBS-containing medium was added followed by centrifugation at 300*g* for 5 min. The cells were then washed with 1× PBS and used for downstream applications, or cell pellets were frozen at −80°C till needed.

### Western blotting

Cervical cancer and control cell lines were harvested by scraping with the addition of lysis buffer. For HIF1α detection, RIPA lysis buffer [1% sodium deoxycholate, 0.1% SDS, 0.15 M NaCl, 0.01 M Na-phosphate, 2 mM EDTA, with freshly added 1 mM PMSF, 2× protease inhibitor cocktail mix (Roche), and 1% NP-40] was used. For E6/E7 detection, 10 mM Tris-HCl, pH 7.5, 50 mM KCl, 2 mM MgCl_2_, and 1% Triton X-100 were used as lysis buffer. Hypoxic or normoxic treatment conditions were maintained during lysis. After 10 min incubation on ice, lysates were centrifuged at 12,000*g* for 5 min. Protein concentrations were determined using DC protein assay (Bio-Rad), and 50 µg (HIF1α detection) or 30 µg (E6/E7 detection) of total protein was loaded on a MiniProtean TGX™ any kD™ gel (Bio-Rad) for separation by SDS-PAGE. Proteins were transferred to PVDF membranes with wet (HIF1α) or semi-dry transfer (E6/E7). Membranes were blocked with 5% dry milk in 1× PBS containing 0.5% Tween-20 and then probed with either anti-HIF1α (#ab179483, Abcam), anti-E6 (#E6-6F4, Euromedex), anti-E7 (#NM2, kind gift from Prof M. Müller, DKFZ), or anti-α-tubulin antibodies (#T5168, Sigma Aldrich) diluted at 1:1,000 overnight at 4°C. Afterwards, membranes were incubated with the respective anti-rabbit (#611-1302, Rockland/Biotrend) or anti-mouse (#115-035-003, Jackson IR) horseradish peroxidase (HRP)-conjugated antibodies diluted at 1:5,000 for 1 h at room temperature. Pierce ECL Western Blotting Substrate (Thermo Scientific) was used for detection of α-tubulin bands, and ECL Prime Western Blot Detection Reagent (GE Healthcare) was used for detecting HIF1α, E6, and E7 bands. Protein bands were visualized with GelDoc™ EZ Imager. Densitometry analysis of Western blot images was done using the ImageJ software (27).

### Detection of APM components by label-free quantification mass spectrometry

Lysates prepared from controls and HPV16-transformed cell lines using RIPA lysis buffer were processed for LC-MS/MS analysis with SP3 (28). Sample acquisition was conducted with an UltiMate™ 3000 RSLCnano (Thermo Fisher Scientific) coupled to an Orbitrap Exploris 480 (Thermo Fisher Scientific) mass spectrometer. Peptides were trapped on a cartridge (Acclaim PepMap 100 Cartridge, C18 100 Å 5 µm, 300 µm × 5mm) for 3 min at a flow rate of 30 µl/min (0.1% TFA in UPLC grade water). Elution and separation of peptides was performed with an analytical column (nanoEase M/Z Peptide BEH C18 column, 130 Å, 1.7 µm, 75 µm × 200 mm) with a flow rate of 300 nl/min and a gradient of solvent A (99.9% water, 0.1% formic acid) and solvent B (80% acetonitrile, 0.1% formic acid, 19.9% water). All solvents were UPLC grade. The gradient set up was 0 min solvent B 2%, 3 min solvent B 2%, 4 min solvent B 5%, 106 min solvent B 38%, 107 min solvent B 95%, 109 min solvent B 95%, 110 min solvent B 2%, and 120 min solvent B 2%. A Nanospray-Flex ion source (Thermo Fisher Scientific) and a Pico-Tip Emitter 360 µm OD × 20 µm ID; 10 µm tip (New Objective) coupled the mass spectrometer and the UPLC. The eluting peptides were ionized in positive mode with 2.2 kV, and the temperature of the ion transfer tube was set to 275°C. Full scan mass spectra were acquired with a scan range of 350–1500 m/z and a resolution of 60,000. Further settings were RF Lens 40%, ACG target 3E6 ions, and maximum injection time 45 ms. Fragmentation of peptides was conducted with data-dependent mode for charge state 2–6. The quadrupole isolation window was set to 1.4 m/z with a collision energy of 26%. Fragmented peptides were recorded with a resolution of 15,000 for a maximum of 1E5 ions (AGC target) or of 22 ms (injection time). Dynamic exclusion was set to 30 s.

MaxQuant software was used for data analysis using an organism-specific database extracted from Uniprot.org under default settings (29). Peptide and protein level identification FDR cutoffs were 0.01 each. Match between runs option was enabled to transfer peptide identifications across Raw files based on accurate retention time and m/z. The MaxLFQ algorithm was used for quantification using a label free quantification method (30). A minimum of two quantified peptides per protein was required for protein quantification. The R-package limma was used for statistical analysis (31). Protein groups with non-zero intensity values in 70% of the samples of at least one of the conditions were used for statistical analysis. For imputation of missing values, the setup with a down-shift of 1.8 standard deviation (SD) and a width of 0.3 SD was adapted from the default parameters of the Perseus software package (32). The *p*-values were adjusted with the Benjamini–Hochberg method for FDR to adjust for multiple testing (33).

### Flow cytometry analysis of HLA-A2 and PD-L1 surface expression

Single-cell suspensions were stained for 20–30 min with anti-HLA-A2-FITC antibody (1:100; #551285, BD Biosciences), Ig-isotype control-FITC antibody (1:100; #401205, BioLegend), anti-PD-L1-BV421 antibody (1:40; #329714, BioLegend), or IgG2b-isotype control-BV421 antibody (1:40; # 400341, BioLegend). After fixation with 1% PFA in PBS for 10–15 min, samples were acquired at a FACS Canto II flow cytometer (BD Biosciences). HLA-A2-positive and HLA-A2-negative gates were set based on isotype control samples. Geometric mean of the FITC or BV421 intensity was assessed. For HLA-A2, the expression fold change in hypoxia relative to normoxia was calculated. Data analysis was performed using FlowJo™ software v10 (BD).

### PBMC isolation

Buffy coat preparations of the blood from anonymous healthy female donors were obtained from DRK Blutspendedienst Mannheim. Material of donors above 40 years of age was used preferably, to increase the probability of prior exposure to HPV. HLA-A2-positive donors were identified by flow cytometry as described before and peripheral blood mononuclear cell (PBMC) isolation was performed for HLA-A2-positive donors using a standard density gradient procedure with Ficoll-Paque™ PLUS (Sigma Aldrich) in Leucosep tubes (Greiner bio-one). The isolated PBMCs were subsequently washed with 1× PBS, and residual erythrocytes were lysed by 5 min incubation with ACK lysis buffer (150 mM NH_4_Cl, 10 mM KHCO_3_, and 0.1 mM EDTA). PBMCs were washed, suspended in human serum with 10% DMSO, and cryopreserved in the gas phase of liquid nitrogen.

### Peptides

Twenty-eight HPV16 E6- and E7-derived peptides previously validated for binding to HLA-A2 were used in this study (**Supplementary Table 2**) (34). For ELISpots, the HLA-A2-restricted human immunodeficiency virus (HIV)-derived peptide Nef_137-145_ (LTFGWCFKL, termed HIV-A2) and the CEF peptide pool (# PA-CEF-001, PanaTecs) were used as negative and positive controls, respectively. For cytotoxicity assays, the HLA-A2-restricted cytomegalovirus (CMV)-derived peptide pp65_495-503_ (NLVPMVATV) and the Epstein–Barr virus (EBV)-derived peptide BMLF1_280-288_ (GLCTLVAML) were used as negative controls. HPV and negative control peptides were synthesized at the GMP unit of the DKFZ.

### ELISpot assay

PBMCs were thawed and one T cell culture per antigen stimulation was set up with 1–2 × 10^6^cells/well in 24-well plates in T cell medium (RPMI-1640 with 10% FBS, 2 mM L-glutamine, 10 mM HEPES, and 1% P/S) supplemented with 10 ng/ml recombinant human Interleukin 7 (rhIL-7; R&D Systems). Each T cell line was stimulated with 10 μg/ml of either one of the 28 HLA-A2-binding HPV16 E6- and E7-derived synthetic peptides, 10 μg/ml HIV-A2 negative control peptide, or 1 μg/ml per peptide of the positive control CEF peptide pool. On day 3 and day 7, cultures were fed with 20 U/ml of recombinant human Interleukin 2 (rhIL-2; PeproTech), with a half medium change at day 7. At day 12, 1–2 × 10^5^ cells/well were transferred to sterile Multiscreen-HA membrane plates (Millipore) coated with 1:500 anti-human IFNγ (clone 1-D1K; Mabtech) in PBS. Cell lines were re-stimulated with respective peptide or CEF peptide pool (six replicates). Concanavalin A (Sigma Aldrich, 2 μg/ml) and DMSO (Sigma Aldrich, 1 μl/ml) (three replicates each) were used as positive and negative controls. After 24 h incubation, cells were discarded and plates were washed with PBS and developed with 1:1,000 biotinylated anti-human IFNγ (clone 7 B6-1-Biotin; Mabtech) in PBS, 1:2,000 Streptavidin-Alkaline Phosphatase solution (Mabtech) in PBS, and filtered substrate (NBT/BCIP; Millipore). Plates were washed with ELISpot wash buffer (PBS with 0.05% Tween 20) between steps. Spots were counted with an automated ImmunoSpot reader (CTL-Immunospot^®^ S6 Ultra-UV). For each well, spot forming units (SFU) per one million cells and the stimulation index (SI) (spot count divided by mean spot count in DMSO-treated control wells) were calculated.

### Generation of peptide-loaded autologous DCs

Human PBMCs were thawed and 1 × 10^7^cells/well were allowed to adhere in six-well plates for 3 h. After adherence of monocytes, supernatant and non-adherent cells were removed and 2 ml of DC medium (DMEM with 10% human serum, 1% L-Glutamine, 1% P/S, and 10 mM HEPES) supplemented with 1,000 U/ml GM-CSF (R&D Systems) and 500 U/ml recombinant human interleukin 4 (rhIL-4; R&D Systems) was added. Three days later, 1ml fresh DC medium, GM-CSF (1,000 U/ml), and rhIL-4 (500 U/ml) were added. On day 6, a maturation cocktail (1,000 U/ml TNF-α, R&D Systems; 10 ng/ml IL-1β, R&D Systems; 10 ng/ml IL-6, R&D Systems; 1 μg/ml PGE2, Cayman Chemical; and 10 U/ml LPS, Invivogen) was added, and on day 8, DCs were harvested by gentle scraping. A total of 10^6^ cells/ml were incubated with 10 µg/ml peptide for 3 h for loading. Finally, peptide-loaded DCs were washed and used as antigen-presenting cells.

### Generation of peptide-specific CD8^+^ T cells

A total of 1 × 10^7^ PBMCs/ml in T cell medium with rhIL-7 (10 ng/ml) were co-cultured with peptide-loaded autologous DCs in ratios of minimum 50:1 to a maximum of 200:1. Every second day, IL-2 (20 U/ml) was added per well, with half medium change when the medium turned acidic. On day 8, cells were stimulated again with peptide-loaded autologous DCs. On day 12, each peptide-specific culture was split into two replicates and each replicate was exposed to either normoxia (20% O_2_, 5% CO_2_) or hypoxia (1% O_2_, 5% CO_2_; InVivo2 workstation, Baker Ruskinn). At day 14, cells were harvested and CD8^+^ T cells were isolated using a MACS CD8^+^ T cell isolation kit (#130-096-495, Miltenyi Biotec). Cell harvesting and MACS sort were performed in normoxic and hypoxic conditions, respectively. T cells were allowed to rest for 30 min before setting up cytotoxicity assays either in normoxic or in hypoxic conditions according to the preceding T cell generation.

### Cytotoxicity assays

Cytotoxicity assays were carried out against the HPV16-positive cervical cell lines CaSki as specific target cells and the HPV-negative cervical cancer cell line C33A as control target cells. The HLA types of these two cell lines closely match (**Supplementary Table 3**). Peptide-specific T cells were generated and expanded in a 14 days pre-culture. Two days before the cytotoxicity assay, the T cell cultures were split in half and exposed to normoxia/hypoxia and the respectively pre-treated T cells were used for the cytotoxicity assay in the same condition. Specific cytotoxicity was assessed in a flow cytometry-based assay as previously published (35). Briefly, peptide-specific CD8^+^ T cells (effector cells) were co-cultured with a 1:1 mixture of specific target cells (CaSki) labeled with 5 µM CFSE (Life Technologies) and HPV-negative control cells (C33A) labeled with 0.25 µM CellTrace Far Red (FR; Life Technologies) in different effector:target (E:T) ratios for 48 h in normoxic or hypoxic conditions. Control wells without T cells were plated under the same conditions to be used for normalization. Cultures were harvested, fixed with 1% PFA, and acquired using a FACS Canto II flow cytometer (BD Biosciences). FlowJo™ software version 10 (BD) was used for data analysis and % specific killing was calculated based on the following formula: 100 − ((% specific target cells with T cells/%control target cells with T cells)/(% specific target cells without T cells/% control target cells without T cells) × 100. Based on the formula, negative killing can be observed in cases where the specific targets proliferate more than the non-specific targets.

### Statistics

Statistical analysis was done using GraphPad Prism v7. The statistical test applied to each dataset is indicated in the respective figure legend.

## Results

### Hypoxia responsiveness of HPV16-transformed cells

For our study, we selected a comprehensive panel of HPV16-tranformed HLA-A2-positive cell lines. It included cell lines obtained from primary cervical tumors or metastases, from patients of different ethnicity, of different duration of cell culture propagation, and transformed with different HPV16 variant lineages (36). We also included two HPV-negative control cell lines, C33A and NOK (**Table 1**).

**Table 1:** HPV16-positive and control cell lines used in the study.

Cell line	Tissue source	Histology	Ethnicity/Country	Year of generation	HPV16 variant (36)
CaSki	Metastasis of cervical carcinoma (small bowel mesentery)	Squamous cell carcinoma	Caucasian	1977 (37)	European (E2)
SNU17	Primary cervical carcinoma	Squamous cell carcinoma	Korea	1997 (38)	European (EA)
SNU703	Primary cervical carcinoma	Squamous cell carcinoma	Korea	1997 (38)	European (EA)
SNU1299	Primary cervical carcinoma	Squamous cell carcinoma	Korea	1997 (38)	Asian American (AA1)
866	Primary	n.a.	Netherlands	2000 (39)	African-2
MRI-H-196	Primary cervical carcinoma	Squamous cell carcinoma, poorly differentiated	Black	n.a.	European (E1)
Marqu	Metastasis	n.a.	Caucasian	2000–2010	European (E1)
C33A	Primary cervical carcinoma	n.a.	Caucasian	1964 (40)	–
NOK	hTert immortalized oral keratinocytes	n.a.	USA	2003 (41)	–

n.a., not available.

**Figure 1:**
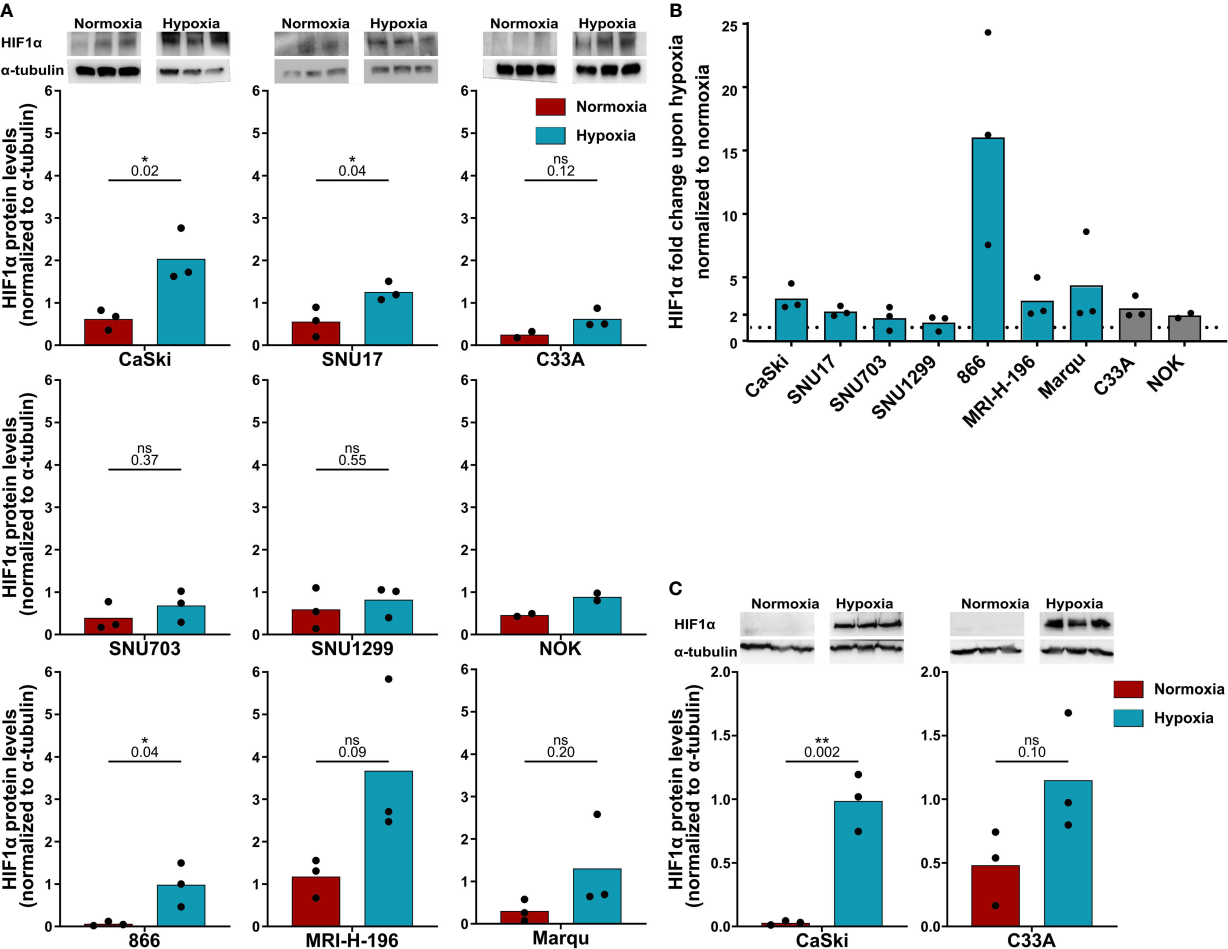
HPV16-transformed cells are responsive to hypoxia. **(A)** Western blot images (upper panel) and quantification (lower panels) of HIF1α levels upon 24 h normoxia (red) and hypoxia (blue) and **(B)** fold change in HIF1α levels under 24 h hypoxia relative to normoxia in HPV16-positive cervical cancer cell lines (blue) and HPV16-negative control cell lines C33A and NOK (gray). The dotted line indicates the HIF1α levels in normoxia, normalized to 1. **(C)** Western blot images (upper panel) and quantification (lower panels) of HIF1α levels upon 48 h normoxia (red) and hypoxia (blue). To quantify HIF1α protein levels, band intensity was normalized to α-tubulin. The mean and individual values of three biological replicates are shown (NOK *n* = 2). Statistical analysis was done using unpaired *t*-test. n.s., non significant (*p*-value >0.05), *p-value ≤ 0.05 and **p-value ≤ 0.01. The *p*-values are mentioned.

First, it was confirmed whether all the cells were indeed responsive to hypoxia by analyzing HIF1α levels by Western blotting (**Figure 1**). Treatment with 24 h hypoxia led to a marked increase in HIF1α levels in the vast majority of cell lines (**Figures 1A, B**). For in-depth analysis, the responsivity of the HPV16-positive CaSki and control C33A cell lines to 48 h hypoxia was investigated as these cell lines were selected as target cells for follow-up assays with 48 h duration. Similar to 24 h hypoxia treatment, 48 h hypoxia treatment increased HIF1α protein levels in both cell lines (**Figure 1C**). Thus, all cell lines were considered suitable for subsequent experiments.

### Decrease in HPV oncoproteins upon hypoxia treatment

**Figure 2:**
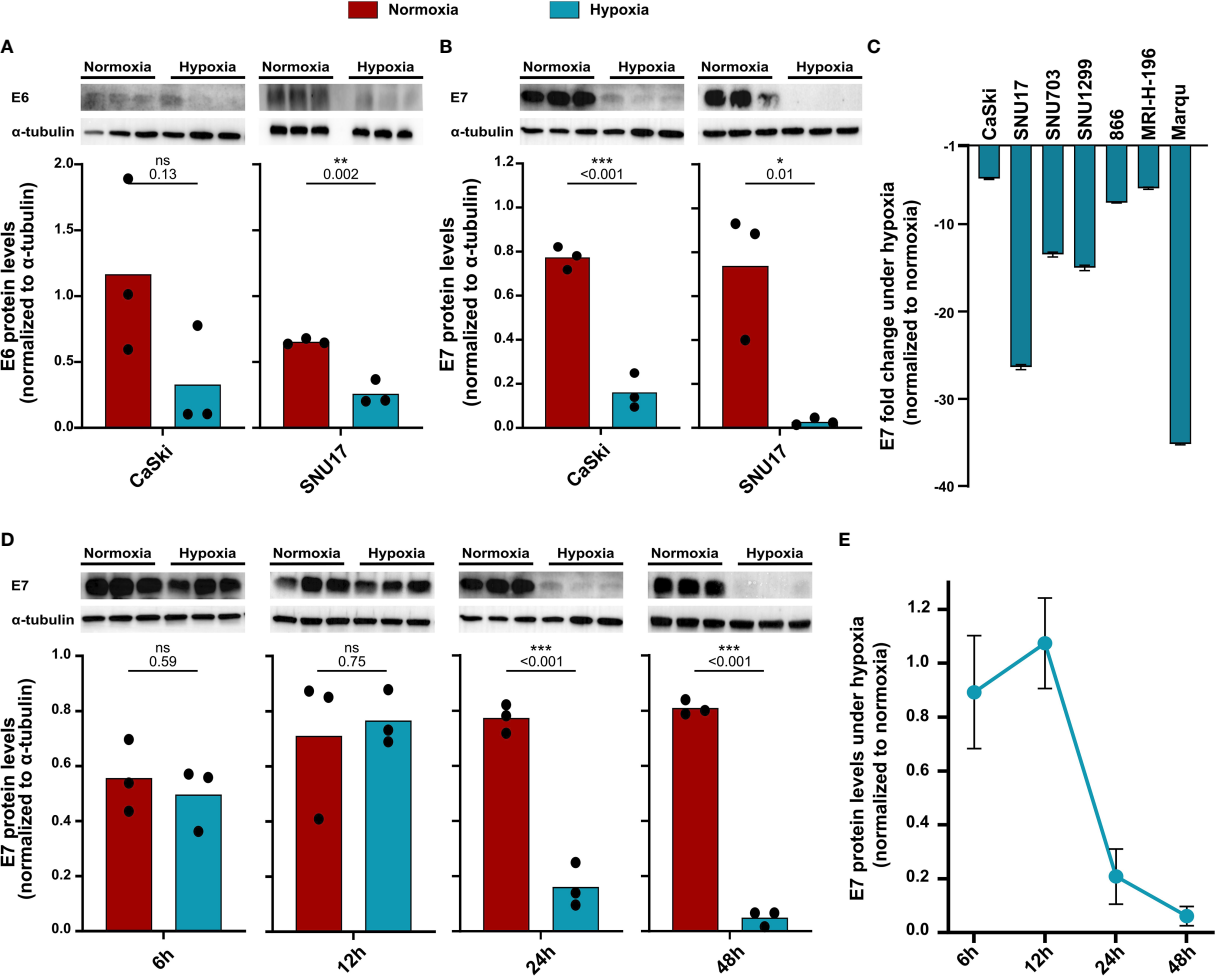
Decrease in protein levels of HPV16 oncoproteins upon hypoxia. Western blots (upper panel) and respective quantification (lower panel) of **(A)** E6 and **(B)** E7 protein levels in HPV16-positive CaSki and SNU17 cells upon 24 h normoxia (red) or hypoxia (blue). **(C)** Fold change in E7 levels upon 24 h hypoxia relative to normoxia in HPV16-positive cervical cancer cell lines. Fold change is given as negative inverse of the ratio between hypoxia and normoxia to indicate reduction of E7 levels. **(D)** Western blots (upper panel) and respective quantification (lower panel) of E7 levels across different time points of treatment with normoxia (red) or hypoxia (blue) in CaSki cells. **(E)** Timeline of fold change in E7 levels in CaSki cells upon hypoxia normalized to normoxia. To quantify E6 and E7 protein levels, band intensity was normalized to α-tubulin. The mean and individual values of three biological replicates are shown. Statistical analysis was done using unpaired *t*-test. n.s., nonsignificant (*p*-value > 0.05), *p-value ≤ 0.05, **p-value ≤ 0.01, and ***p-value ≤ 0.001. The *p*-values are mentioned.

Previous studies have shown that the main HPV oncoproteins E6 and E7 are decreased in HPV16-transformed CaSki upon hypoxia treatment (23). Since these proteins are the main targets for immunotherapeutic approaches, we assessed E6 and E7 protein levels in our cell line panel. In CaSki and SNU17, a decrease in E6 levels upon 24 h hypoxia was observed (**Figure 2A**). E7 protein levels were also found to be decreased upon 24 h hypoxia in CaSki and SNU17 (**Figure 2B**), as well as in the other HPV16^+^ cell lines (**Figure 2C**). Cultivation of CaSki in hypoxic conditions with high glucose (4.5 g/L) medium prevented downregulation of E7 (**Supplementary Figure 1**), demonstrating that the observed hypoxia-mediated E7 repression in CaSki is glucose sensitive. This supplements published data, where this effect has already been described for several other HPV^+^ cell lines (23). We went on to investigate the timeline of the decrease in the expression of E7 in the model cell line CaSki. No decrease in E7 protein levels was observed for up to 12 h of hypoxia treatment. However, E7 protein levels decreased clearly upon 24 h hypoxia treatment, and this decrease was sustained until 48 h (**Figures 2D, E**). Thus, our results verify existing literature about the effect of hypoxia on the HPV16 E6 and E7 oncoproteins and extend results to a total of seven HPV16-transformed cell lines.

### No effect of hypoxia on the APM in HPV16-transformed cells

**Figure 3:**
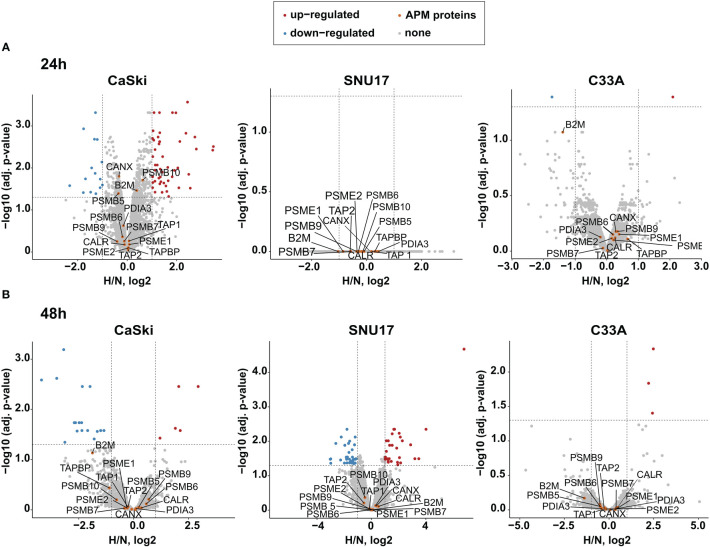
Effect of hypoxia on the APM in target cells. Volcano plots depicting the –log10 (adjusted *p*-values) *vs*. the log2 fold change upon hypoxia *vs*. normoxia (H/N, log2) of the whole proteome upon **(A)** 24 h hypoxia treatment and **(B)** 48 h hypoxia treatment of CaSki, SNU17, and C33A cells. Protein expression is either upregulated (red), downregulated (blue), or unchanged (gray). The APM proteins of interest (orange) are annotated. Thresholds (dashed lines) are at log fold change of ±1 and a *p*-value cutoff of 0.05.

Given that the oncoprotein E7 is still expressed for up to 24 h of hypoxia (**Figures 2D, E**), it needs to be determined whether epitopes from this protein can still be presented under hypoxia. Thus, we next investigated the effect of hypoxia on the APM in our panel of HPV16-transformed cells and controls. Because of its diverse effects on cellular pathways, expression of most proteins is regulated by hypoxia. Therefore, normalization to a single common housekeeping gene or protein is not appropriate. To achieve an unbiased analysis, we used label-free quantification (LFQ) MS. This technique uses intrinsic normalization and is thus best suited to study the effects of hypoxia on the APM (30). We analyzed numerous APM components, reflecting a peptide’s path from protein degradation to MHC surface presentation, namely, proteasomal subunits (PSME 1 and 2, PSMB 5–10); transporter proteins (TAP 1 and 2); enzymes (ERAP1 and 2); chaperones involved in peptide loading into the MHC (calreticulin, calnexin, and PDIA3); and the surface presentation molecules (HLA I and Beta-2-microglobulin). Volcano plots depicting the fold change in the whole proteome upon hypoxia *vs*. the significance of the change for CaSki, SNU17, and C33A can be seen in **Figure 3**, for 24 h (A) and 48 h (B) hypoxia treatment. In these cells, the expression of the APM proteins is unchanged upon 24 h and 48 h hypoxia. Similarly, 24 h hypoxia did not show any effect on the APM components in the other cell lines of our panel (**Supplementary Figure 2**). Taken together, hypoxia did not significantly change APM components in cervical cancer cell lines.

**Figure 4:**
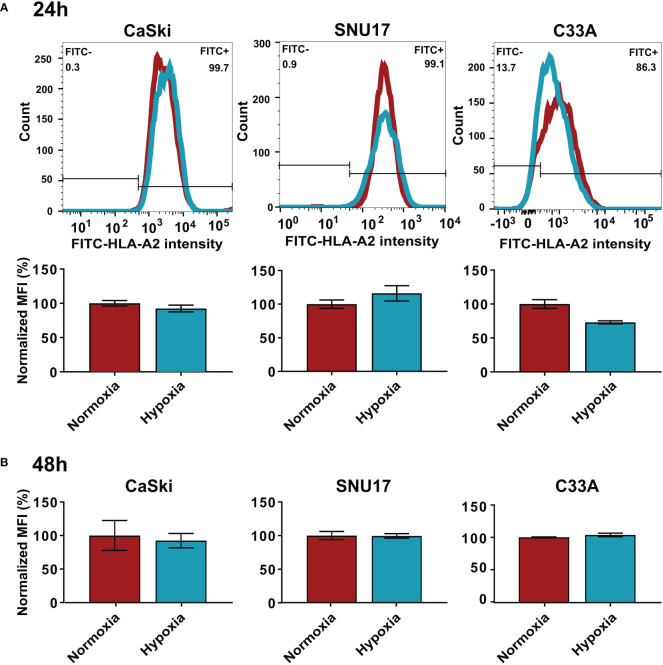
Effect of hypoxia on HLA-A2 levels in target cells. Representative histograms and bar graphs of HLA-A2 (FITC) intensities upon 24 h **(A)** and 48 h **(B)** hypoxia treatment of CaSki, SNU17, and C33A. The histograms of normoxic (red) and hypoxic (blue) examples are overlaid. Bar graphs depict the mean MFI (mean fluorescence intensity) of biological triplicates of normoxic and hypoxic cells ± SD normalized to normoxia, with normoxic MFI set to 100%.

While MS analyzes the total expression levels of the protein, it does not address whether the cell surface expression of HLA I molecules is changed upon hypoxia. Thus, we performed flow cytometry to assess surface expression of HLA-A2, the common HLA I molecule in our cell line panel, in normoxia- and hypoxia-treated cells. We observed that hypoxia treatment for 24 h or 48 h did not significantly change the surface expression levels of HLA-A2 in HPV16-transformed CaSki and SNU17 cells and HPV-negative C33A cells (**Figures 4A, B**) or any of the other HPV16-positive cell lines or controls (**Supplementary Figure 3**). This substantiates the MS findings, and suggests that peptides from residual E6 and E7 proteins could still be surface-presented to the immune system.

### Differential effects of hypoxia on CD8^+^ T cell-mediated cytotoxicity

**Figure 5:**
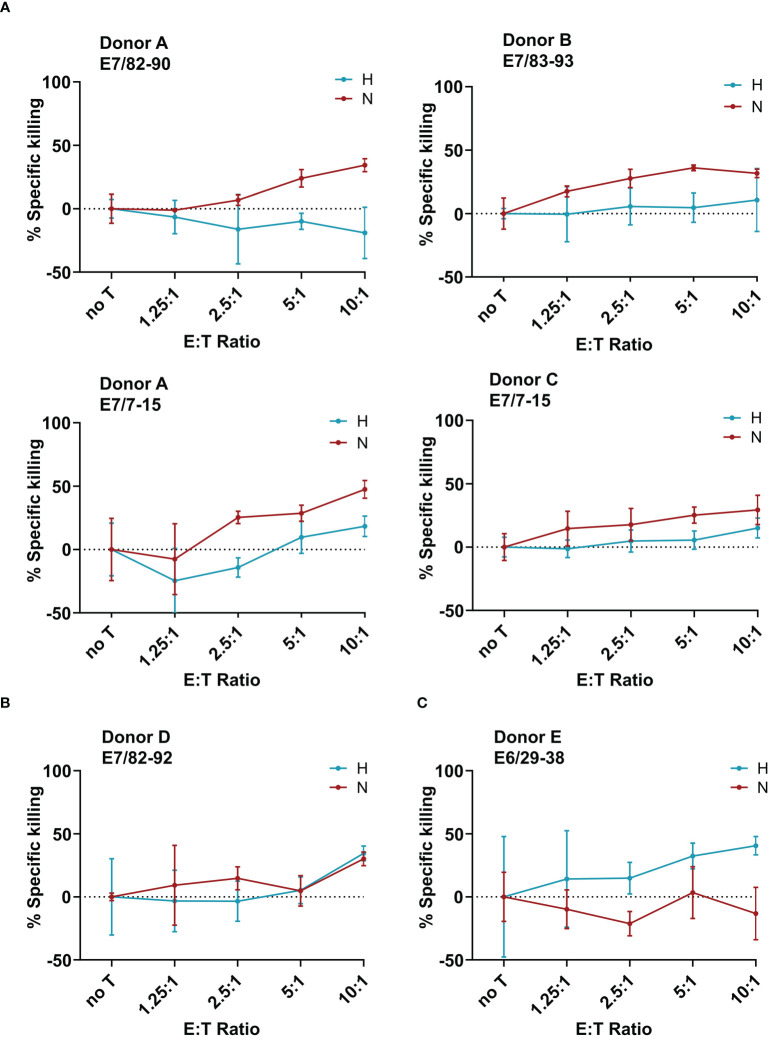
Effect of hypoxia on killing of CaSki by E6- and E7-specific CD8^+^ T cell lines. Calculated specific killing of HPV16-positive CaSki cells relative to HPV-negative C33A control cells at different ratios of CD8^+^ T cells to target cells (E:T ratio) is shown. The specific killing of each epitope-specific T cell line was assessed in hypoxia (blue) and normoxia (red). We observed **(A)** decreased specific killing of CaSki cells under hypoxia, **(B)** no difference in killing in normoxia and hypoxia, and **(C)** increased killing of specific target cells in hypoxic conditions. Negative specific killing is a result of CaSki cells outgrowing C33A control cells. The mean of three technical replicates ± SD (error bars) is shown.

After having observed a downregulation of the HPV oncoproteins, and no effect on the APM, the next goal was to investigate the effect of hypoxia on the killing of target cells by cytotoxic T cells. To generate HPV16 E6- and E7-derived peptide-specific CD8^+^ T cells, healthy donors were screened for memory responses to a pre-selected repertoire of 28 HLA-A2-binding peptides derived from HPV16 E6 and E7 using ELISpot assays. The ELISpot results are summarized in **Supplementary Figure 4**. Some peptides, such as E7_82-90_, were observed to be immunogenic in multiple donors, while others, such as E6_25-33_, showed strong immunogenicity in only one donor. Most of the responses were observed against HPV16 E7-derived peptides. For selected peptides that induced immune memory responses, peptide-specific T cell lines were generated. A flow-cytometry-based co-culture assay (Vital FR) was used for cytotoxicity assessment. The working principle of the Vital FR assay, representative flow cytometry plots, and an exemplary result are shown in **Supplementary Figure 5**. Negative controls are shown in **Supplementary Figure 6**. Analysis of the specific killing of CaSki cells in hypoxic relative to normoxic conditions demonstrated varying results. Cytotoxicity was observed to be either reduced (**Figure 5A**), unaffected (**Figure 5B**), or enhanced (**Figure 5C**). As T cells in these experiments were derived from different donors and raised against different peptides, these differences seem to be donor- and/or peptide-specific.

**Figure 6:**
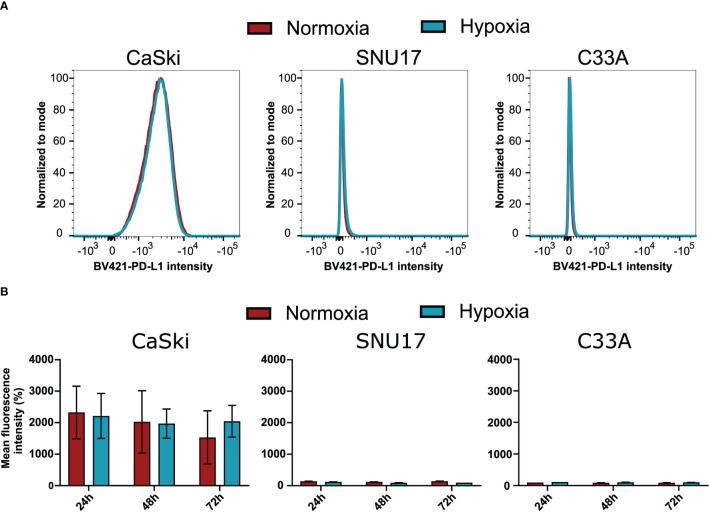
Effect of hypoxia on PD-L1 surface expression levels in target cells. **(A)** Representative histograms of PD-L1 (BV421) upon 24 h hypoxia treatment of CaSki, SNU17, and C33A. The histograms of normoxic (red) and hypoxic (blue) examples are overlaid. **(B)** Bar graphs show the mean MFI (mean fluorescence intensity) of triplicates of normoxic and hypoxic cells ± SD after 24 h, 48 h, and 72 h incubation. For CaSki, the results of triplicates from three independent experiments (each with triplicates) are shown.

To assess whether the observed effects of hypoxia on T cell cytotoxicity are influenced by changes of the expression of inhibitory checkpoint molecule PD-L1 on the target cells, we investigated the surface expression of PD-L1 in hypoxia and normoxia by flow cytometry. SNU17, SNU1299, MRI-H-196, and C33A showed no or extremely low surface expression of PD-L1 in normoxic conditions, while CaSki, SNU703, 866, Marqu, and NOK showed detectable PD-L1 surface expression. Irrespective of the initial expression level, treatment with hypoxia for 24 h, 48 h, and 72 h had no or only minor effects on the surface expression of PD-L1 in our panel of cell lines (**Figure 6** and **Supplementary Figure 7**). This suggests that the effect of hypoxia on cytotoxic T cell-mediated killing of cervical cancer cells is not affected by regulation of this checkpoint inhibition pathway.

Our results suggest that hypoxia influences cytotoxic T cells, and can lead to suppressed or enhanced killing of cervical cancer target cells. This effect seems to be independent of the influence of hypoxia on the target cells, as APM components and PD-L1 expression were observed to be unchanged, and decreased E7 expression would only explain decreased killing.

## Discussion

Hypoxia is a universal feature of the TME of solid tumors, including cervical cancer (23). Enhancement of tumor invasiveness, metastasis, immunosuppression, and immune escape under hypoxic conditions make hypoxia a major challenge for the development of successful immunotherapies (42). The importance of investigating the influence of the TME is highlighted by the marked improvements in therapies for advanced cervical cancer observed upon combination of the anti-angiogenic drug bevacizumab with prevalent chemotherapeutic protocols (43, 44). However, it is important to gain a full understanding of the TME’s pleiotropic effects. Therefore, we wanted to gain insights into the effects of hypoxia, a key regulator in the TME, on HPV16-transformed human cells and immune responses against them.

Unfortunately, there are no suitable murine HPV-induced cervical cancer models for this purpose. The most commonly used mouse tumor models, such as TC-1 or C3, were generated by transduction or transfection with HPV16 genes, respectively (45, 46). However, they originate from lung and embryonic C57Bl/6 cells, not allowing for an accurate study of the TME of naturally HPV16-transformed cells. Additionally, they are most commonly studied in a subcutaneous setting, which does not resemble the mucosal environment. Only a few recent studies use tumor models in an orthotopic setting (47). Finally, and most importantly, murine cells do not present clinically relevant HLA-restricted epitopes, preventing direct examination of relevant peptides. The recently developed tumor models by our group in MHC-humanized A2.DR1 mice indeed present human epitopes to the immune system (48) and are even located in the vaginal mucosa of mice (47). However, so far, they have not been studied for hypoxia, and as these models depend on tumor cell transplantation, the TME most likely will not resemble those of naturally growing tumors. Thus, we decided to perform our study in a panel of human HPV16-transformed cells, maintained under conditions mimicking one aspect of the TME, namely, the oxygen levels.

We observed a decreased expression of HPV oncoproteins in HPV16-transformed cells upon 24 h hypoxia treatment in a comprehensive panel of HPV16-transformed cells. This confirms existing literature on the effect of hypoxia on HPV16 E6 and E7 expression and expands this knowledge to a broader set of cell lines (48). Our results additionally demonstrate that cervical cancer cell lines differing in tissue source (primary carcinoma/metastases), ethnicity/country of origin, duration of cell culture propagation, and transforming HPV16 variant lineages (**Table 1**), all respond similarly to hypoxia. Since the viral proteins E6 and E7 are the targets of choice for many immunotherapies (4, 7), a decrease in their expression under hypoxic conditions of the TME is of concern. Investigating the timeline of decrease of E7 protein expression upon hypoxia, it was observed here that in CaSki cells, E7 protein levels are not decreased by 12 h of hypoxia treatment, and even after up to 24 h of hypoxia, some residual protein was detected by Western blotting (**Figures 2D, E**). As long as the E6 and E7 proteins are still expressed, epitopes derived from these proteins can be presented on the cell surface *via* HLA I. However, hypoxia has been reported to dysregulate some components of the APM *in vitro* and *in vivo* in sarcoma and pulmonary mouse tumors (21). Therefore, regulation of the APM by hypoxia could potentially affect the surface presentation of antigens. Our results, however, showed no effect of 24 h hypoxia treatment on the APM in any of the HPV16-transformed cell lines, as well as the HPV-negative control cells (**Figure 3A** and **Supplementary Figure 2**). Even 48 h of hypoxia did not affect the expression of any of the APM components in CaSki and SNU17 (**Figure 3B**). The lack of regulation of the APM by hypoxia in any of the cell lines is surprising, given the pleiotropic effects of hypoxia. However, by choosing LFQ analysis, it was possible to investigate the regulation of the APM proteins under hypoxia compared to the whole cell proteome. Thus, we ascertained that there is indeed no effect of hypoxia on the APM.

For all these experiments, 24- and 48 h time points—and no longer hypoxia duration—were analyzed as we did not observe any important additional changes at 48 h compared to 24 h. It is, however, possible that cells exposed to chronic hypoxia adjust their functions further, which we do not address in this study. On the other hand, it is important to note that cervical cancers have been described to have cycling hypoxia, i.e., intermittent periods of hypoxia followed by reoxygenation (24, 49). These cycles are expected to be in the range of the time points analyzed herein.

Next, we confirmed the effect of hypoxia on cell surface presentation of HLA-A2 by flow cytometry (**Figure 4** and **Supplementary Figure 3**). In contrast to previous reports about increase (19, 20) or decrease (21) in HLA class I (HLA I) surface levels upon hypoxia, we did not observe a significant change in HLA-A2 expression upon 24 h hypoxia treatment in any of the cell lines. This suggests that the presentation of epitopes from E6 and E7 in hypoxic HPV16-transformed cells might not be perturbed, as long as enough source protein is expressed. It has been reported that the effects of hypoxia on HLA I expression are more pronounced on cells in 3D culture systems as compared to 2D cultured counterparts in murine sarcoma cells (21). Since our experiments were carried out in 2D cultures of cervical cancer cell lines, it would be interesting to investigate these effects in a cervical cancer 3D culture or organoid system, which has been recently developed (50, 51). It is also important to note that our study focused on HLA-A2, the most frequently expressed HLA I allotype in the Caucasian population. Thus, it remains possible that hypoxia affects HLA I expression in an allotype-dependent manner.

The next question was to assess the effect of hypoxia on HPV-specific T cells. HPV oncoproteins have low expression levels in infected cells, which increases only upon cell transformation into cancer (5). Despite low expression of the oncoproteins, most HPV infections are cleared by effective T cell immunity (52). This implies sufficient protein expression for successful priming of epitope-specific T cells, which is also supported by the presence of memory T cell responses against HPV16 E6 and E7-derived peptides observed in healthy donors [(53–55) and screened by us (**Supplementary Figure 4**)]. The success associated with therapeutic HPV vaccines (7) or T cells transgenic for HPV epitope-specific TCRs (56) causing lesion or tumor regression further indicates sufficient HPV epitope presentation on target cells to make them detectable by cytotoxic T cells.

When assessing the effect of hypoxia on HPV-specific T cells, we observed that hypoxia mediated decreased, unchanged, and increased cytotoxicity against HPV16^+^ CaSki cells in a peptide- and donor-specific manner in our small panel of HPV16-specific T cell lines. These opposing results are in line with existing contradictory literature about the effect of hypoxia on cytotoxic T cells in the context of different murine tumors (17, 18). In our experiments, four out of six T cell lines demonstrated reduced specific killing of CaSki cells under hypoxia, which is in line with reduced expression of HPV oncoproteins. However, since our results were obtained using only one cervical cancer target cell line, and we also observed unchanged or enhanced killing under hypoxia, this suggests that possibly the T cells rather than the tumor cells behave differentially under hypoxia. An alternative explanation could be that the repertoire of peptides presented on the surface of hypoxic CaSki cells is altered as compared to normoxic CaSki. However, given that we did not observe any effect of hypoxia on the APM, this explanation seems unlikely.

Hypoxia has also been reported to increase PD-L1 expression, which is a known checkpoint inhibition protein (57). However, our results show that PD-L1 is unaffected by hypoxia in the panel of cervical cancer cell lines, indicating that this checkpoint inhibition pathway is not involved in the differential effects of hypoxia on CD8^+^ T cell-mediated cytotoxicity towards cervical cancer cells.

As mentioned above, hypoxia in the cervical cancer microenvironment is not static but characterized by intermittent reoxygenation, caused by poor vasculature (23). This condition, which is known as cycling hypoxia (24, 49), most likely prevents complete downregulation of E6 and E7 and would thereby allow sufficient presentation of epitopes on the cell surface *in vivo*. The diversity and the fluidity of the conditions within the TME suggest that glucose concentrations are also variable within tumors. As the downregulation of HPV oncogenes was seen to be dependent on low glucose levels [ (23) and **Supplementary Figure 1**], this would also result in residual E6/E7 expression under hypoxia. Taken together, our data indicate that a hypoxic TME might not influence the success of a therapeutic vaccination. However, the combination of a therapeutic vaccine with measures that either alleviate hypoxia or reduce tumor burden and thereby hypoxic areas in the tumor might be beneficial.

## Data availability statement

The mass spectrometry proteomics data have been deposited to the ProteomeXchange Consortium *via* the PRIDE partner repository with the dataset identifier PXD032217. All other data generated or analyzed during this study are included in this published article. Raw data are available from the corresponding author upon reasonable request.

## Ethics statement

Ethical review and approval was not required for the study of human participants in accordance with the local legislation and institutional requirements. Written informed consent from the patients/participants was not required to participate in this study in accordance with the national legislation and the institutional requirements.

## Author contributions

NM and AR conceptualized the study, and AR supervised the whole project. NM, KW, CÖ, NV, and SF conducted experiments. NM, KW, CÖ, NV, JF, SF, and MB contributed to data analysis. NM, KW, MB, and AR worked on data visualization and validation. NM, KW, and AR wrote the paper. All authors contributed to the article and approved the submitted version.

## Funding

The project was carried out with DKFZ core funding.

## Acknowledgments

We would like to thank Prof. Martin Müller (DKFZ) for the kind gift of the anti-E7 antibody. We thank Prof. Felix Hoppe-Seyler and his group for their help with the hypoxia chamber experiments. Prof. Jeroen Krijgsveld, Karim Aljakouch, and Nicolas Palacio-Escat are thanked warmly for their help with the LFQ analysis. We would like to acknowledge the contribution of Martin Schneider, DKFZ Proteomics core facility, for help with the MaxQuant data analysis, and the DKFZ Flow cytometry core facility. Alexandra Klevenz and Rebecca Köhler are acknowledged for excellent technical assistance.

## Conflict of interest

The authors declare that the research was conducted in the absence of any commercial or financial relationships that could be construed as a potential conflict of interest.

## Publisher’s note

All claims expressed in this article are solely those of the authors and do not necessarily represent those of their affiliated organizations, or those of the publisher, the editors and the reviewers. Any product that may be evaluated in this article, or claim that may be made by its manufacturer, is not guaranteed or endorsed by the publisher.

## Abbreviations

HPV, human papillomavirus; HLA, human leukocyte antigen; APM, antigen processing and presentation machinery; ECM, extracellular matrix; HIF, hypoxia-inducible factor; MHC, major histocompatibility complex; MS, mass spectrometry; PBMCs, peripheral blood mononuclear cells; TME, tumor microenvironment.
