# Supplementary material for: Effects of hypoxia on antigen presentation and T cell-based immune recognition of HPV16-transformed cells
Source: Front Immunol. 2022 Oct 21;13:918528. doi: 10.3389/fimmu.2022.918528 (PMC9634485; doi:10.3389/fimmu.2022.918528)
Supplement: Supplementary file 1 [file DataSheet_1.docx]

Supplementary Material

## Effects of hypoxia on antigen presentation and T cell-based immune recognition of HPV16-transformed cells

Supplementary Methods

**Supplementary Table 1. Cell lines and corresponding media**

| **Cell line** | **Standard medium** | **Low glucose medium** |
| --- | --- | --- |
| NOK | Keratinocyte SFM + Epidermal Growth Factor (5ng/ml) + Bovine Pituitary Extract (50µg/ml) | Keratinocyte SFM + Epidermal Growth Factor (5ng/ml) + Bovine Pituitary Extract (50µg/ml) |
| C33A | DMEM (4.5 g/L) + FCS (10%) + P/S (1%) + L-Glutamine (1%) | DMEM (1 g/L) + FCS (10%) + P/S (1%) +  L-Glutamine (1%) |
| MRI-H-196 | DMEM (4.5 g/L) + FCS (10%) + P/S (1%) + L-Glutamine (1%) | DMEM (1 g/L) + FCS (10%) + P/S (1%) +  L-Glutamine (1%) |
| Marqu | DMEM (4.5 g/L) + FCS (10%) + P/S (1%) + L-Glutamine (1%) | DMEM (1 g/L) + FCS (10%) + P/S (1%) +  L-Glutamine (1%) |
| CaSki | DMEM (4.5 g/L) + FCS (10%) + P/S (1%) + L-Glutamine (1%) | DMEM (1 g/L) + FCS (10%) + P/S (1%) +  L-Glutamine (1%) |
| SNU17 | RPMI-1640 (1 g/L) + FCS (10%) + P/S (1%) + L-Glutamine (1%) + HEPES (1%) | RPMI-1640 (1 g/L) + FCS (10%) + P/S (1%) + L-Glutamine (1%) + HEPES (1%) |
| SNU703 | RPMI-1640 (1 g/L) + FCS (10%) + P/S (1%) + L-Glutamine (1%) + HEPES (1%) | RPMI-1640 (1 g/L) + FCS (10%) + P/S (1%) + L-Glutamine (1%) + HEPES (1%) |
| SNU1299 | RPMI-1640 (1 g/L) + FCS (10%) + P/S (1%) + L-Glutamine (1%) + HEPES (1%) | RPMI-1640 (1 g/L) + FCS (10%) + P/S (1%) + L-Glutamine (1%) + HEPES (1%) |
| 866 | F12-medium (75%) + DMEM (4.5 g/L) (25%) + FBS (5%) +P/S (1%)  + hydrocortisone (0.4 mg/ml)+ insulin (5 mg/ml), + cholera toxin (0.01 mg/ml) + adenine (24.2µg/ml) + EGF (0.1 mg/ml) | F12-medium (75%) + DMEM (1 g/L) (25%) + FBS (5%) +P/S (1%)  + hydrocortisone (0.4 mg/ml)+ insulin (5 mg/ml), + cholera toxin (0.01 mg/ml) + adenine (24.2µg/ml) + EGF (0.1 mg/ml) |

FCS: Fetal calf serum; SFM: Serum free medium; P/S: Penicillin/Streptomycin, EGF: Epidermal Growth Factor

**Supplementary Table 2. List of peptides used in this study**

| **Peptide** | **Sequence** |
| --- | --- |
| HPV16 E6_18-26_ | KLPQLCTEL |
| HPV16 E6_18-28_ | KLPQLCTELQT |
| HPV16 E6_25-33_ | ELQTTIHDI |
| HPV16 E6_28-38_ | TTIHDIILECV |
| HPV16 E6_29-38_ | TIHDIILECV |
| HPV16 E6_34-44_ | ILECVYCKQQL |
| HPV16 E6_52-60_ | FAFRDLCIV |
| HPV16 E7_7-17_ | TLHEYMLDLQP |
| HPV16 E7_11-18_ | YMLDLQPE |
| HPV16 E7_11-19_ | YMLDLQPET |
| HPV16 E7_11-20_ | YMLDLQPETT |
| HPV16 E7_11-21_ | YMLDLQPETTD |
| HPV16 E7_12-19_ | MLDLQPET |
| HPV16 E7_12-20_ | MLDLQPETT |
| HPV16 E7_66-74_ | RLCVQSTHV |
| HPV16 E7_76-86_ | IRTLEDLLMGT |
| HPV16 E7_77-86_ | RTLEDLLMGT |
| HPV16 E7_77-87_ | RTLEDLLMGTL |
| HPV16 E7_80-90_ | EDLLMGTLGIV |
| HPV16 E7_81-90_ | DLLMGTLGIV |
| HPV16 E7_81-91_ | DLLMGTLGIVC |
| HPV16 E7_82-90_ | LLMGTLGIV |
| HPV16 E7_82-91_ | LLMGTLGIVC |
| HPV16 E7_82-92_ | LLMGTLGIVCP |
| HPV16 E7_83-93_ | LMGTLGIVCPI |
| HPV16 E7_84-93_ | MGTLGIVCPI |
| HPV16 E7_85-93_ | GTLGIVCPI |
| HPV16 E7_86-93_ | TLGIVCPI |

**Supplementary Table 3. HLA-types of CaSki and C33A**

|  | **HLA-A** | **HLA-A (supertype*)** | **HLA-B** | **HLA-B (supertype*)** |
| --- | --- | --- | --- | --- |
| **CaSki** | 02:01 | 03:01 (A03) | 07:02 | 37:01 (B44) |
| **C33A** | 02:01 | 11:01 (A03) | 07:02 | 44:02 (B44) |

*Sidney J, Peters B, Frahm N, Brander C, Sette A, HLA class I supertypes: A revised and updated classification. BMC Immunol. 2008;9:1.

Supplementary Results


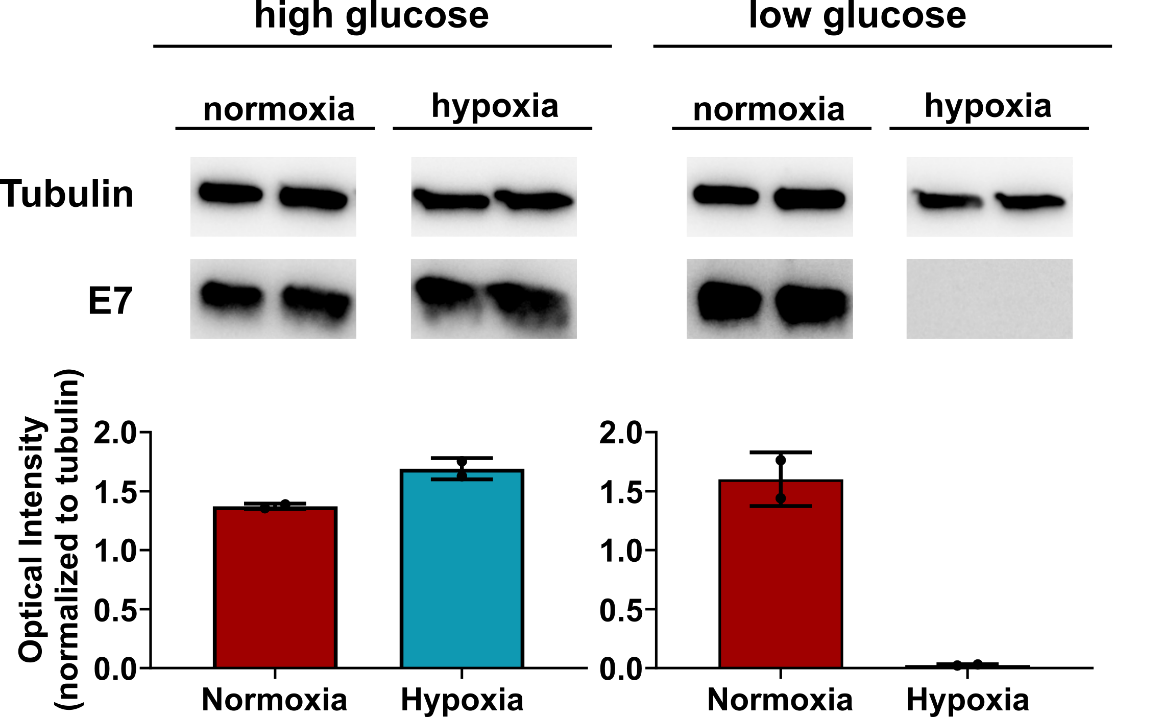


**Supplementary Figure 1. High glucose concentration prevents downregulation of E7 in hypoxic conditions.** A) Western blot images (upper panel) and quantification (lower panels) of E7 levels upon normoxia (red) and hypoxia (blue) in high and low glucose medium. To quantify E7 protein levels, band intensity was normalized to α-tubulin. The mean and individual values of biological replicates are shown.


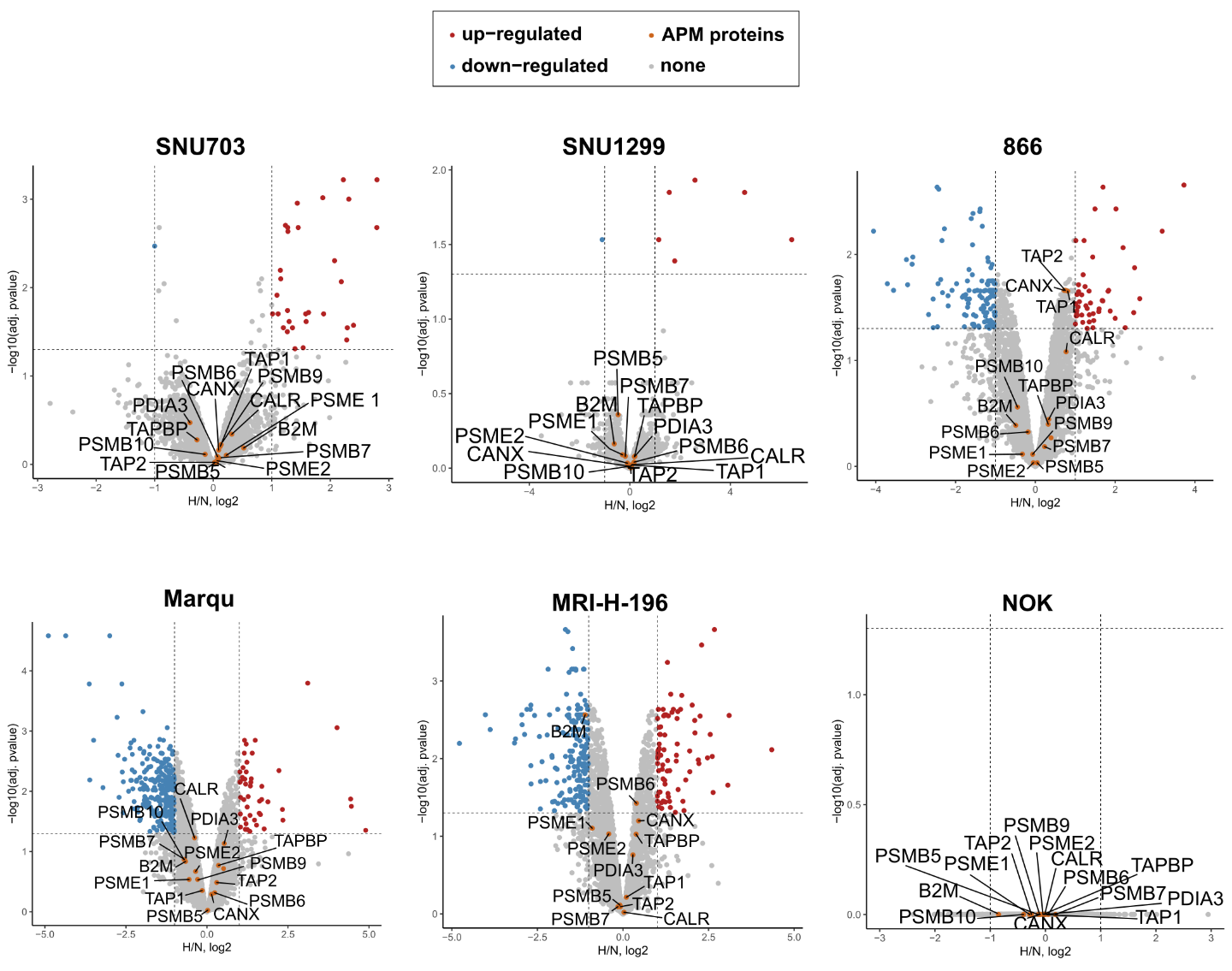


**Supplementary Figure 2. Effect of hypoxia on the APM in the remaining cell lines of the HPV16-positive and -negative cell line panel.** Volcano plots depicting the –log10 (adjusted p values) vs the log2 fold change upon hypoxia (H/N, log2) of the whole proteome upon 24h hypoxia treatment in five HPV16-transformed cell lines and HPV-negative NOK cells. Protein expression is either upregulated (red), downregulated (blue) or unchanged (grey). The APM proteins of interest (orange) are annotated. Thresholds (dashed lines) are at log fold change of +/-1 and a p value cutoff of 0.05.


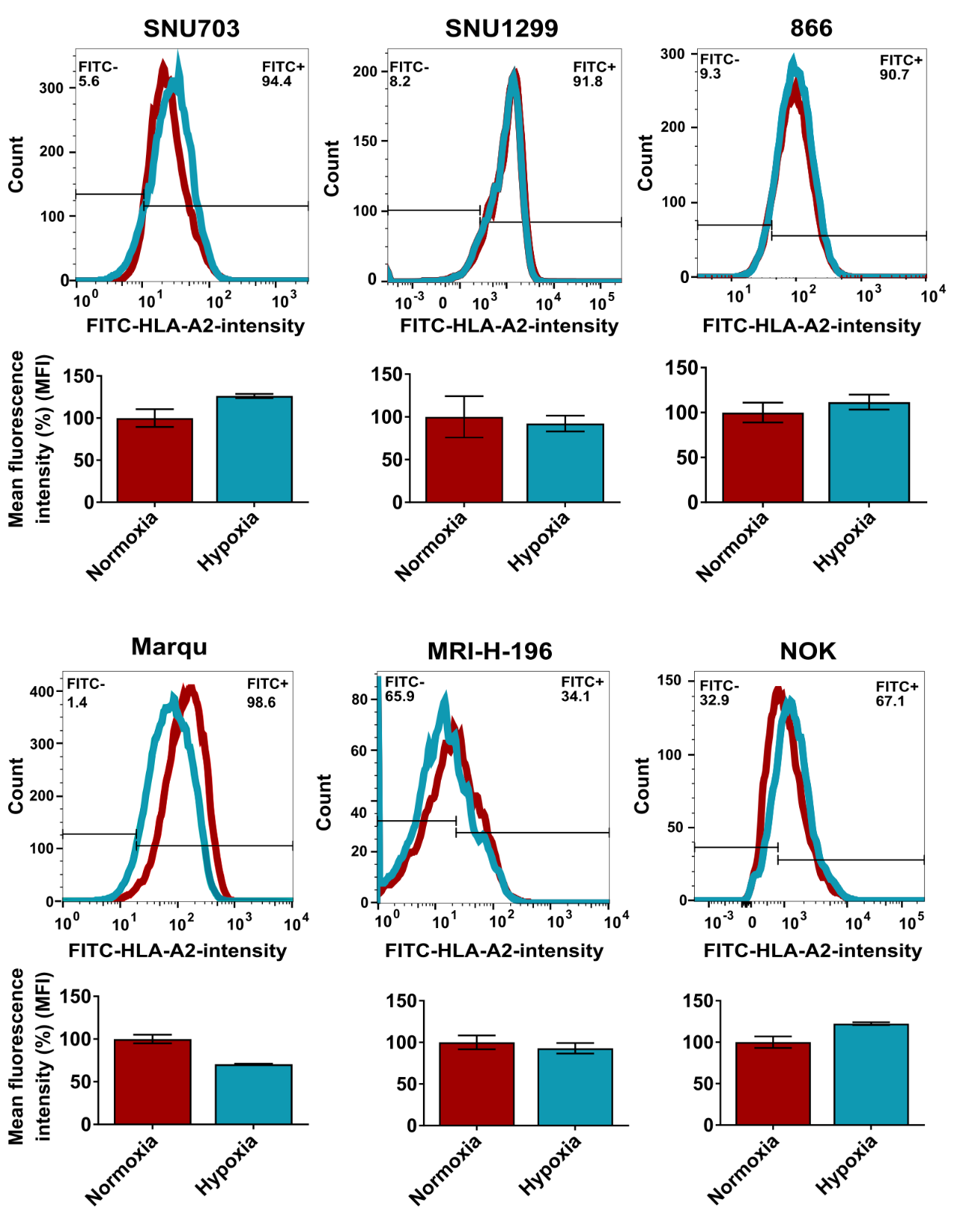


**Supplementary Figure 3. Effect of hypoxia upon HLA-A2 levels in the remaining cell lines of the HPV16-positive and -negative cell line panel.** Representative histograms and bar graphs of HLA-A2 (FITC) upon 24h hypoxia treatment of the remaining HPV16-transformed cells and HPV-negative NOK cells. The normoxic (red) and hypoxic (blue) samples are overlayed in the histograms. Bar graphs depict the mean MFI (mean fluorescence intensity) of triplicates of normoxic and hypoxic cells +/- SD normalized to normoxia, with normoxic MFI set to 100%.


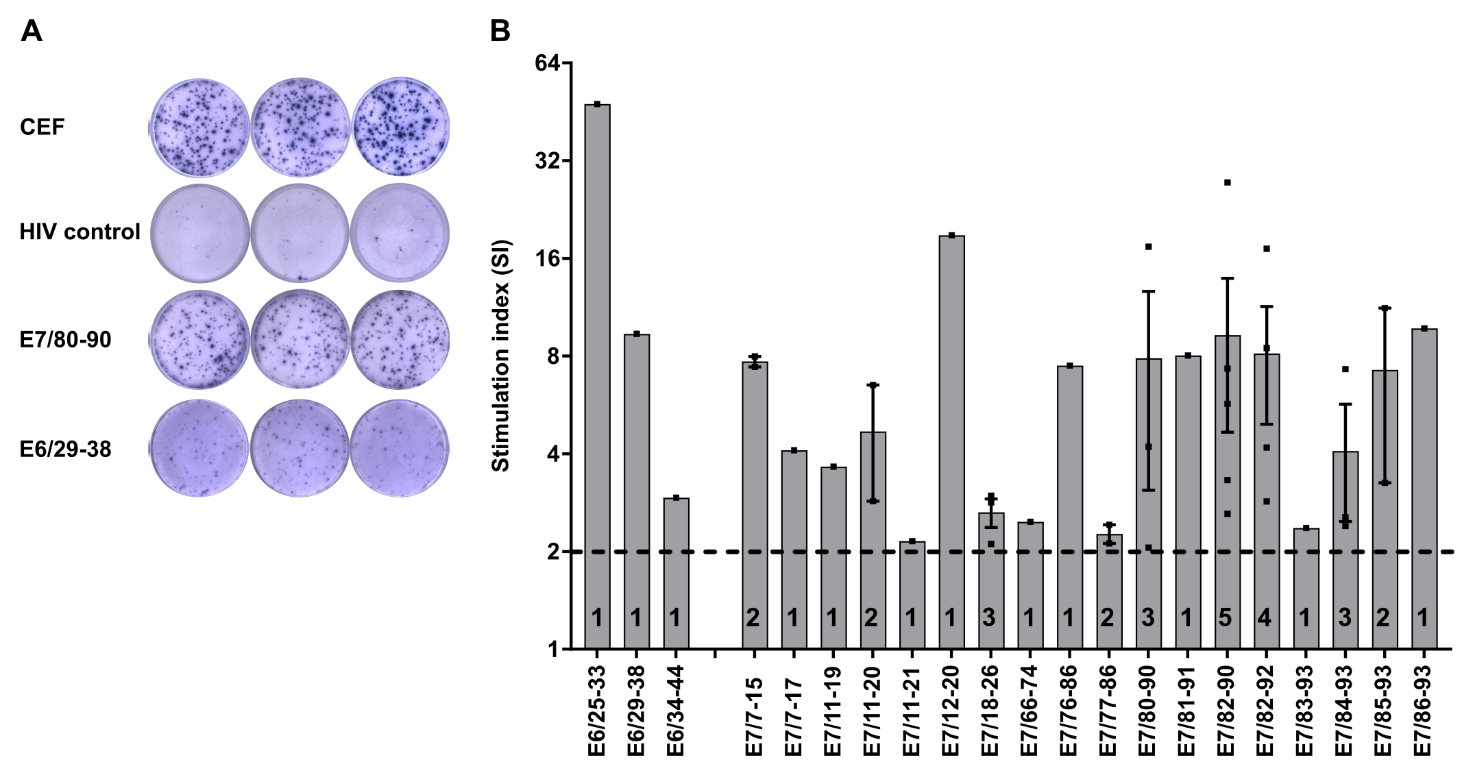


**Supplementary Figure 4. Screening donors for memory responses against HPV16 E6- and E7-derived peptides.** A) Representative ELISpot wells depicting the positive control (CEF), negative control (HIV-A2), a strong response towards a peptide (E7/80-90), and a weakly immunogenic peptide (E6/29-38). B) Stimulation index (SI; ratio of SFU peptide and SFU DMSO control) of all HPV16 E6- and E7-derived HLA-A2 binding peptides that had an SFU ≥ 200/one million cells. Mean SI (+SD) is depicted for individual peptides. The numbers within the bars represent the number of donors showing responses against a given peptide.


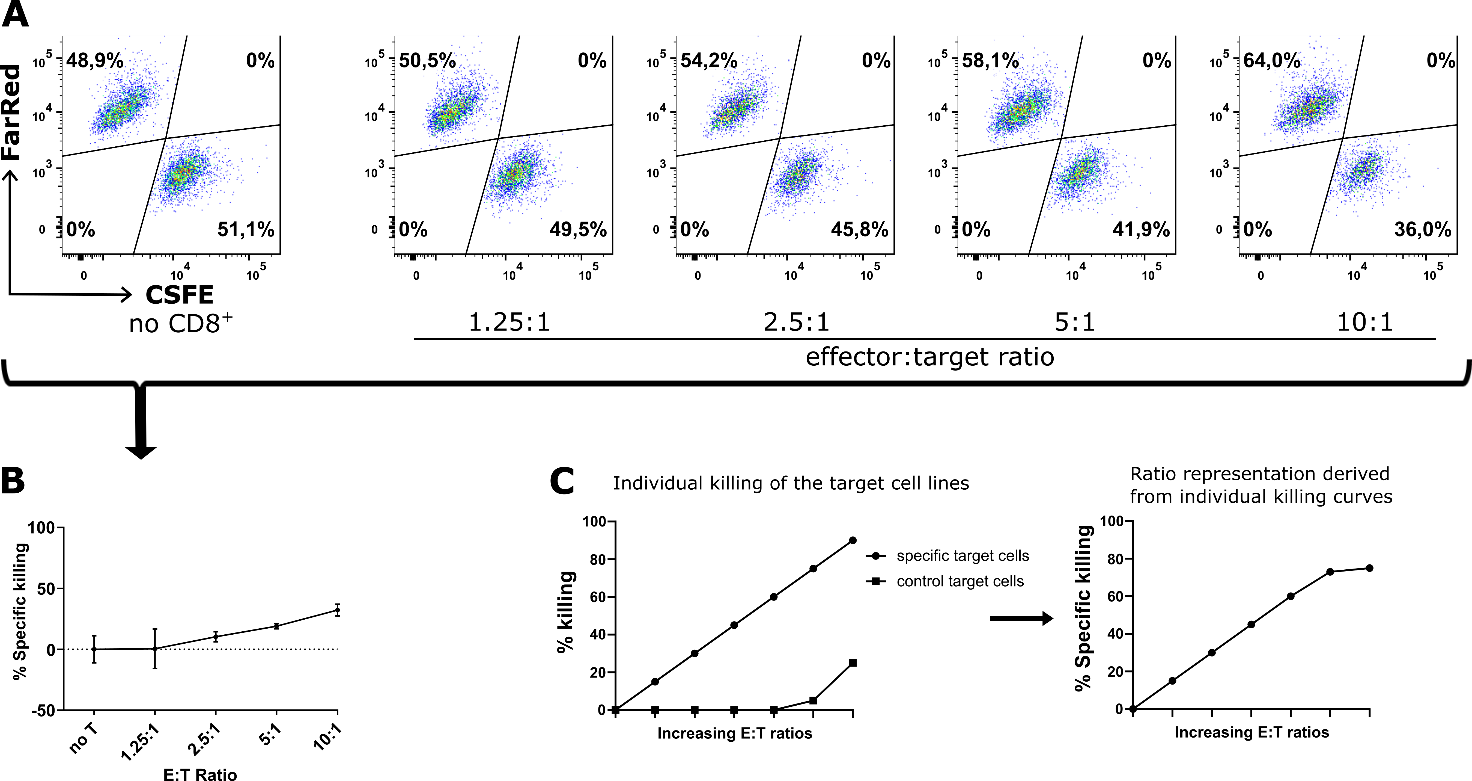


**Suppl. Figure 5.** Working principle of the VitalFR flow cytometry-based cytotoxicity assay. Effector cells (in this case isolated CD8^+^ T cells from T cell expansion cultures) were added at different effector-to-target (E:T) ratios to wells containing a 1:1 mixture of specific target cells labeled in green (here: CaSki cells, CFSE-labeled) and control target cells labeled in red (here: C33A, FarRed (FR)-labeled). 48 h after addition of CD8^+^ T cells, the cellular contents of the experimental wells are harvested, fixed and analyzed via flow cytometry.

(A) Exemplary FACS plots. With increasing E:T ratios, more specific target cells (CFSE-labelled) are killed and their relative amount decreases in the FACS plots, while the absolute number of control target cells (FR-labelled) remains the same, thus their relative amount increases. (B) The ratio of specific target/control target cells is plotted as “% specific killing”. (C) Schematic visualization of how the ratio representation (% specific killing) derives from the individual killing of the specific and unspecific target cells. An increase with increasing E:T ratios is indicative of specific killing of the specific target cells compared to the control target cells. Unspecific killing of the control target cells at high E:T ratios causes a flattening of the “% specific killing” curve.


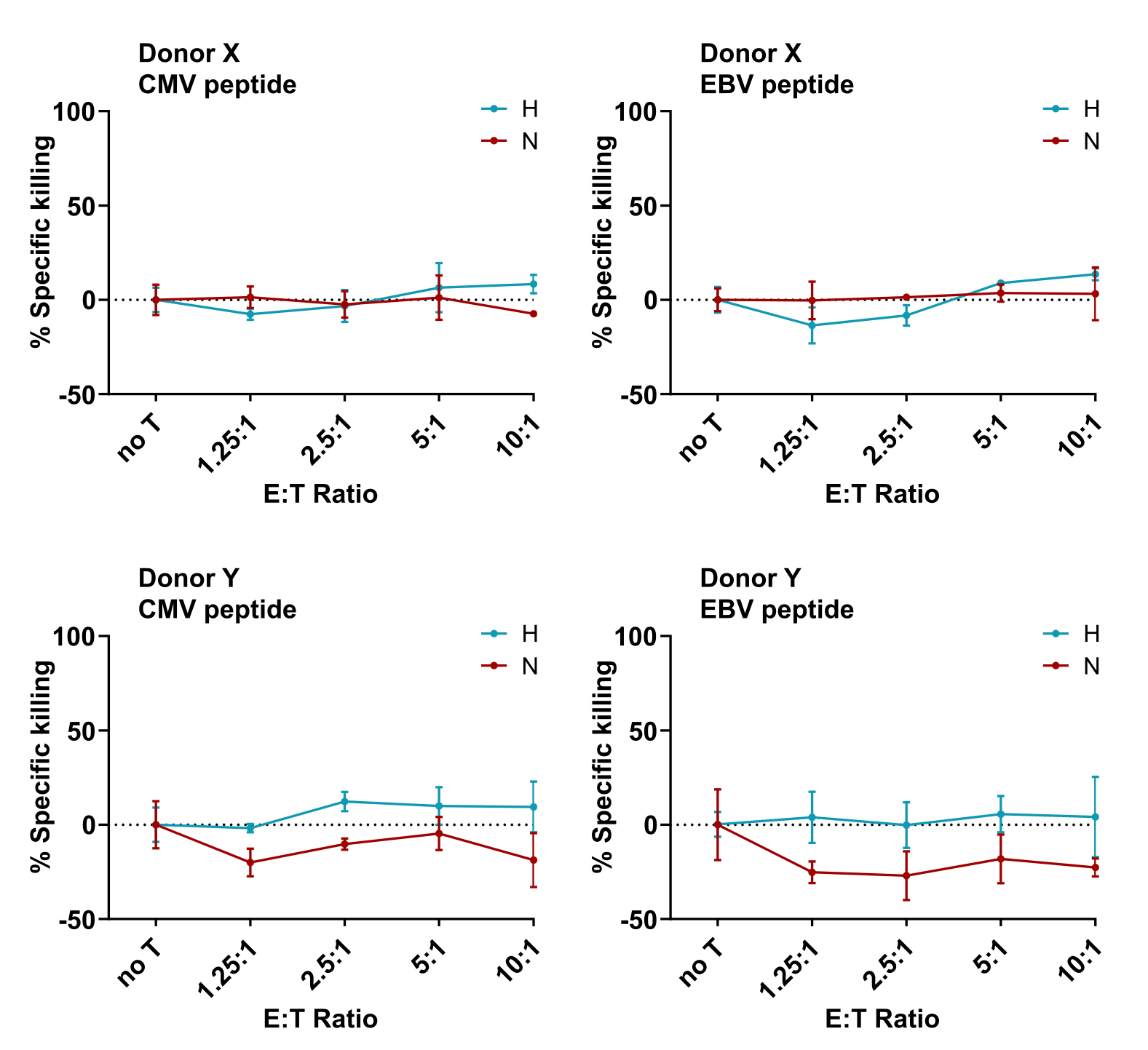


**Suppl. Figure 6. No specific killing of cervical cancer cells by CMV or EBV-specific CD8^+^ T cell lines in normoxia and hypoxia.** Calculated specific killing of HPV16-positive CaSki cells relative to HPV-negative C33A control cells at different ratios of CD8^+^ T cells to target cells (E:T ratio) is shown. The specific killing of each epitope-specific T cell line was assessed in hypoxia (blue) and normoxia (red). Negative specific killing is a result of CaSki cells outgrowing C33A control cells. The mean of three technical replicates +/- SD (error bars) is shown.


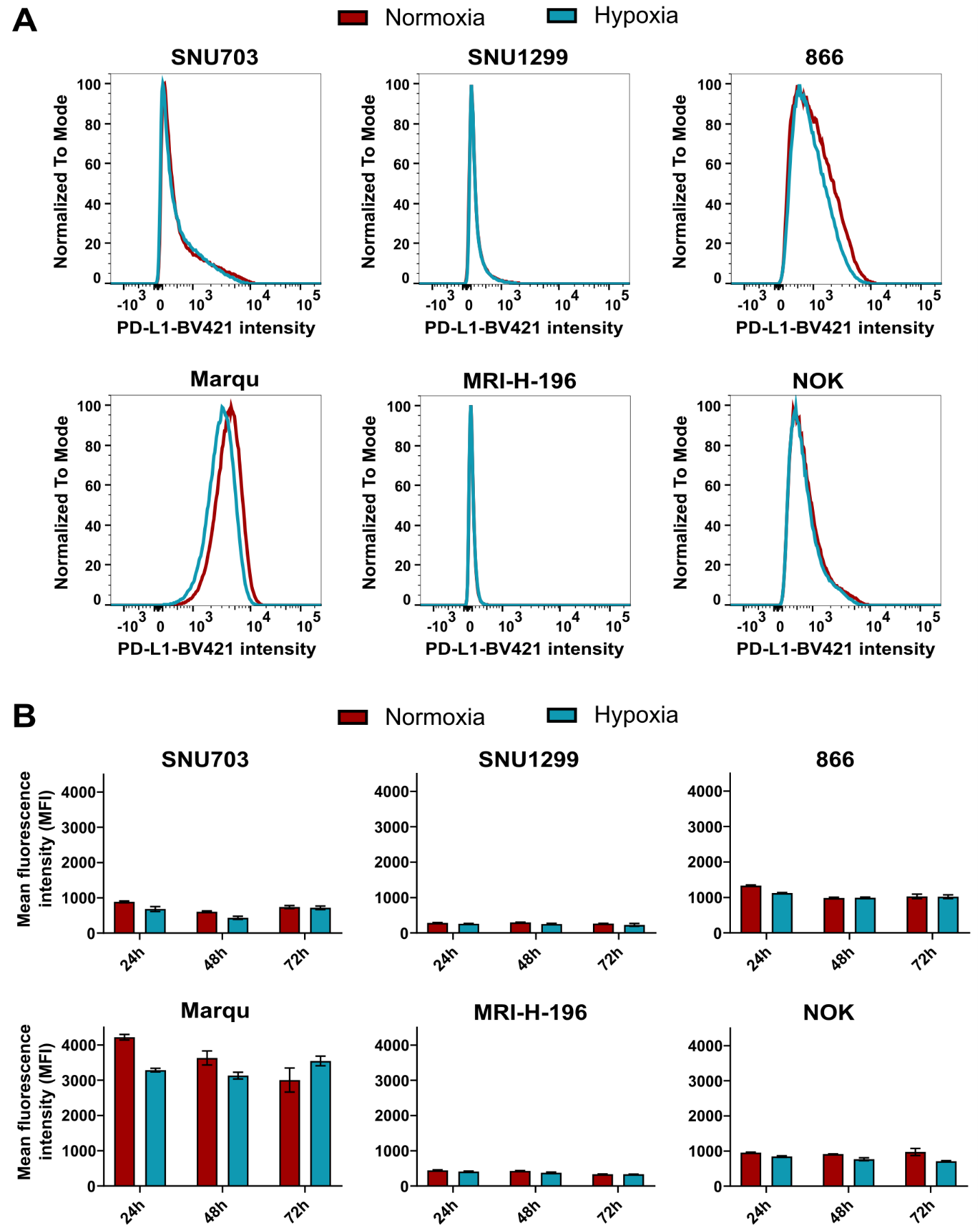


**Supplementary Figure 7.** **Effect of hypoxia on PD-L1 surface expression levels in remaining cell lines.** (A) Representative histograms of PD-L1 (BV421) upon 24h hypoxia treatment of SNU703, SNU1299, 866, Marqu, MRI-H-196 and NOK. The histograms of normoxic (red) and hypoxic (blue) examples are overlayed. (B) Bar graphs show the mean MFI (mean fluorescence intensity) of technical triplicates of normoxic and hypoxic cells +/- SD after 24h, 48h and 72h incubation.
